# Proposal for Sets of ^77^Se NMR Chemical Shifts in Planar and Perpendicular Orientations of Aryl Group and the Applications

**DOI:** 10.1155/BCA/2006/79327

**Published:** 2006-11-28

**Authors:** Satoko Hayashi, Waro Nakanishi

**Affiliations:** Department of Material Science and Chemistry, Faculty of Systems Engineering, Wakayama University, 930 Sakaedani, Wakayama 640-8510, Japan

## Abstract

The orientational effect of *p*-YC_6_H_4_ (Ar) on *δ*(Se) is elucidated for ArSeR, based on experimental and theoretical investigations. Sets of *δ*(Se) are proposed for **pl** and **pd** employing 9-(arylselanyl)anthracenes (**1**) and 1-(arylselanyl)anthraquinones (**2**), respectively, where Se–*C*_*R*_ in ArSeR is on the Ar plane in **pl** and perpendicular to the plane in **pd**. Absolute magnetic shielding tensors of Se (*σ*(Se)) are calculated for ArSeR (R = H, Me, and Ph), assuming **pl** and **pd**, with the DFT-GIAO method. Observed characters are well reproduced by the total shielding tensors (*σ* ^t^(Se)). The paramagnetic terms (*σ* ^P^(Se)) are governed by *σ* ^P^(Se)_xx_ + *σ* ^P^(Se)_yy_, where the direction of n_P_(Se) is set to the *z*-axis. The mechanisms of the orientational effect are established both for **pl** and **pd**. Sets of *δ*(Se: **1**) and *δ*(Se: **2**) act as the standards for **pl** and **pd**, respectively, when *δ*(Se) of ArSeR are analyzed based on the orientational effect.

## INTRODUCTION

^77^ Se NMR spectroscopy is one of powerful tools to study selenium compounds [1–20], containing bioactive materials [21–24]. ^77^ Se NMR chemical shifts (*δ*(Se)) are sharply sensitive to the structural changes in selenium compounds. Therefore, they are widely applied to determine the structures [6–20] and to follow up the reactions of selenium compounds [1–10]. The *δ*(Se) values have been analyzed variously. The substituent effect is employed when the effect of the electronic conditions around Se on *δ*(Se) is examined in *p*-YC_6_H_4_SeR perturbed by Y, for example [6–20]. Some empirical rules and/or classifications between structures and *δ*(Se) are proposed [6–20], however, it is not so easy to predict *δ*(Se) from the structures with substantial accuracy. Some important rules would be behind the observed values. Plain rules, founded on the theoretical background, are necessary to analyze the structures of selenium compounds based on *δ*(Se) and also to understand the origin of *δ*(Se) [25].

**Chart 1:**
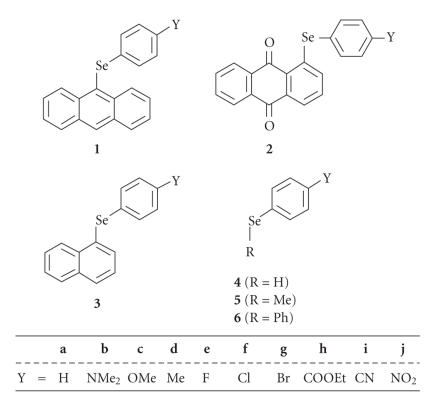


We have pointed out the importance of the orientational effect on *δ*(Se) of *p*-YC_6_H_4_SeR, for the better understanding of *δ*(Se) of ArSeR in a uniform manner [19, 20, 25]. To establish the orientational effect, we present two series of *δ*(Se) for *p*-YC_6_H_4_SeR whose structures (conformers) are fixed to planar (**pl**) and perpendicular (**pd**) conformers for all Y examined, under the conditions [26, 27]. (The nonplanar and nonperpendicular conformer (**np**) is also important in some cases, such as the **CC** conformer in 1,8-(MeZ)_2_C_10_H_6_ (Z = S and Se) [28–33].) (The importance of relative conformations in the substituent effects between substituents and probe sites is pointed out.) The Se−C_R_ bond in ArSeR is on the Ar plane in **pl** and perpendicular to the plane in **pd**. 9-(Arylselanyl)anthracenes (*p*-YC_6_H_4_SeAtc: **1**) and 1-(arylselanyl)anthraquinones (*p*-YC_6_H_4_SeAtq: **2**) are the candidates for **pl** and **pd**, respectively: Y in **1** and **2** are H (**a**), NMe_2_ (**b**), OMe (**c**), Me (**d**), F (**e**), Cl (**f**), Br (**g**), COOEt (**h**), CN (**i**), and NO_2_ (**j**) (see Chart 1). Conformers of the 9-anthracenyl (9-Atc) and 1-anthraquinonyl (1-Atq) groups in **1** and **2** are represented by the type **A** (**A**), type **B** (**B**), and type **C** (**C**) notation, which is proposed for 1-(arylselanyl)naphthalenes (*p*-YC_6_H_4_SeNap: **3**) [14–16, 19, 20, 26]. The structure of **1** is **A** for 9-Atc and **pl** for Ar, which is denoted by **1** (**A**: **pl**). That of **2** is **B** for the 1-Atq and **pd** for **Ar** (**2** (**B**: **pd**)). The series of *δ*(Se) in **1** (*δ*(Se: **1**)) and *δ*(Se: **2**) must be typical for **pl** and **pd**, respectively.

Recently, the reliability of the calculated absolute magnetic shielding tensors (*σ*) is much improved [34–39] and the calculated tensors for Se nuclei (*σ*(Se)) are demonstrated to be useful in usual selenium compounds [28–33].^1^ As shown in (1), the total absolute magnetic shielding tensor (*σ^t^*) is decomposed into diamagnetic (*σ^d^*) and paramagnetic (*σ^p^*) contributions [40, 41].^2^ *σ^p^* contributes predominantly to *σ^t^* in the structural change of selenium compounds. Magnetic shielding tensors consist of three components, as exemplified by *σ^p^* in (2) as the following:

*σ^t^* = *σ^d^* + *σ^p^*, (1)

*σ^p^* = (*σ^p^_xx_* + *σ^p^_yy_* + *σ^p^_zz_*)/3. (2)

Quantum chemical (QC) calculations are performed on ArSeH (**4**), ArSeMe (**5**), and ArSePh (**6**) to understand the orientational effect based on the theoretical background (see Chart 1). The conformations are fixed to **pl** and **pd** in the calculations. The gauge-independent atomic orbital (GIAO) method [42–46] is applied to evaluate *σ*(Se) at the DFT (B3LYP) level. Mechanisms of the orientational effect are explored for **pl** and **pd** based on the magnetic perturbation theory on the molecules.

After the establishment of the orientational effect of aryl group in *p*-YC_6_H_4_SeR, together with the mechanism, *δ*(Se) of some aryl selenides are plotted versus *δ*(Se: **1**) and/or *δ*(Se: **2**). The treatment shows how *δ*(Se) of aryl selenides are interpreted based on the orientational effect. And it is demonstrated that the sets of *δ*(Se: **1**) and *δ*(Se: **2**) give a reliable guideline to analyze the structures of *p*-YC_6_H_4_SeR based on *δ*(Se).

## RESULTS

**Scheme 1:**
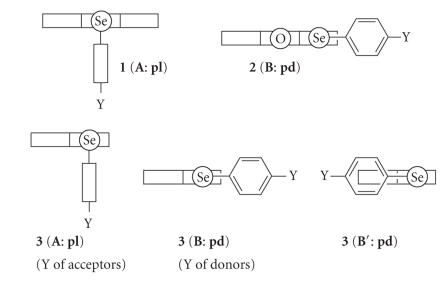
Structures of **1** and **2**, together with those of **3**.

The structures of all members of **1** and **2** are predicted to be **1** (**A**: **pl**) and **2** (**B**: **pd**), respectively [25]. The results are supported by the X-ray crystallographic analysis carried out for **1** and **2**, containing **1c** and **2a** and the QC calculations for **1a** and **2a**, together with the spectroscopic measurements, although not shown. Scheme 1 illustrates **1** (**A**: **pl**) and **2** (**B**: **pd**), together with some conformers of **3**.

Table 1 shows *δ*(Se: **1**) and *δ*(Se: **2**), measured in chloroform-*d* solutions (0.050 M) at 213 K, 297 K, and 333 K.^3^ *δ*(Se: **1a**) and *δ*(Se: **2a**) are given from MeSeMe and *δ*(Se: **1**) and *δ*(Se: **2**) are from **1a** and **2a**, respectively, (*δ*(Se)_SCS_). To examine the temperature dependence in **1**, *δ*(Se: **1**)_SCS_ at 297 K (*δ*(Se: **1**)_SCS, 297 K_) and *δ*(Se: **1**)_SCS, 333 K_ are plotted versus *δ*(Se: **1**)_SCS, 213 K_. Table 2 collects the correlations, where the correlation constants (*a* and *b*) and the correlation coefficients (*r*) are defined in the footnote of Table 2 (entries 1 and 2). *δ*(Se: **2**)_SCS, 297 K_ and *δ*(Se: **2**)_SCS, 333 K_ are similarly plotted versus *δ*(Se: **2**)_SCS, 213 K_. Table 2 also contains the correlations (entries 3 and 4). The *a* values for **1** are smaller than those for **2**. The results show that the temperature dependence in **1** is larger than that of **2**, although both correlations are excellent (*r* > 0.999). The results show that **2** (**B**: **pd**) are thermally very stable and other conformers are substantially negligible in the solution for all Y examined. **1** (**A**: **pl**) must also be predominant in solutions, although **1** (**A**: **pl**) would not be thermally so stable, relative to the case of **2** (**B**: **pd**).

**Scheme 2:**
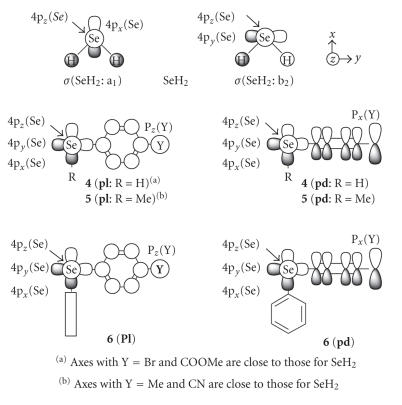
Axes and some orbitals of **4**–**6**, together with those of SeH_2_.

Scheme 2 shows the axes and some orbitals of **4**–**6**, together with SeH_2_. While the *x*-axis of SeH_2_ is in the bisected direction of ∠HSeH, the Se−H and Se−C bonds of MeSeH are almost on the *x*- and *y*-axes, respectively, although not shown. Axes of **4**–**6** are close to those in MeSeH in most cases. Since ∠CSeX (X = H or C) in **4**–**6** are about 95°, 98°, and 101°, respectively, the Se−C and Se−H bonds deviate inevitably from the axes to some extent. Axes are rather similar to those of SeH_2_ for **4** (**pl**) with Y = Br and COOMe and **5** (**pl**) with Y = Me and CN.^4^

Structures of **4**–**6** in **pl** and **pd** are optimized employing the 6-311+G(3df) basis sets for Se and the 6-311+G(3d,2p) basis sets for other nuclei of the Gaussian 03 program [47].^5^ Calculations are performed at the density functional theory (DFT) level of the Becke three parameter hybrid functionals with the Lee-Yang-Parr correlation functional (B3LYP). Absolute magnetic shielding tensors of Se (*σ*(Se)) are calculated based on the DFT-GIAO method [42–46], applying on the optimized structures with the method. Tables 3–5 collect *σ^t^*(Se), *σ^d^*(Se), *σ^p^*(Se), and the components of *σ^p^*(Se), *σ^p^*(Se)_*xx*_, *σ^p^*(Se)_*yy*_, and *σ^p^*(Se)_*zz*_ for **4**–**6** bearing various substituents Y in **pl** and **pd**, respectively.^6^

Relative shielding constants of **A** (*σ^t^*_rel_(Se: **A**)) are calculated for **4**–**6** according to (3), using *σ^t^*(Se: MeSeMe) (= 1650.4 ppm). *σ^t^*_rel_(Se: **A**)_SCS_ are calculated similarly. Table 1 also contains *σ^t^*_rel_(Se: **A**) of **4a**–**6a** and *σ^t^*_rel_(Se: **A**)_SCS_ for **4**–**6**,

*σ^t^_rel_*(Se : **A**)
= −{*σ^t^*(Se : **A**) − *σ^t^*(Se : MeSeMe)} (**A** : **n**(**pl**), **n**(**pd**)). (3)

**Chart 2:**
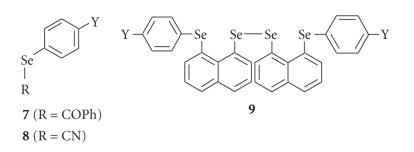


Table 6 shows *σ*(Se)_SCS_ of *p*-YC_6_H_4_SeCOPh (**7**) [13], *p*-YC_6_H_4_SeCN (**8**) [8], and bis[8-(arylselanyl)naphthyl] 1,1′-diselenides (**9**) [15, 16], together with **5** [11, 19] and **6** [15, 16] (see Chart 2). The values are plotted versus *δ*(Se: **1**)_SCS_ and/or *δ*(Se: **2**)_SCS_ to explain the *δ*(Se) based on the orientational effect of the aryl groups.

## DISCUSSION

### Characters in *δ*(Se: **1**) and *δ*(Se: **2**)

The structures of all members of **1** and **2** are confirmed to be **1** (**A**: **pl**) and (**B**: **pd**), respectively, (see Scheme 1) [25]. The nature of *δ*(Se: **1**) must be the results of **1** (**A**: **pl**), where n_*p*_(Se) is parallel to the *π*(C_6_H_4_Y-*p*). Characteristic points in *δ*(Se: **1**)_SCS_ are summarized as follows.

1. Large upfield shifts (−23 ppm to −6 ppm) are observed for Y = NMe_2_, OMe, and Me and large downfield shifts (17 ppm to 33 ppm) are for Y = COOEt, CN, and NO_2_, relative to Y = H.
2. Moderate upfield shift (−3 ppm) is observed for Y = F.
3. Small downfield shifts (2 ppm) are for Y = Cl and Br: the three points corresponding to Y = H, Cl, and Br are found very close with each other.

The characters of *δ*(Se: **2**)_SCS_ are very different from those of *δ*(Se: **1**)_SCS_. The characteristics must be the reflection of **2** (**B**: **pd**), where n_*p*_(Se) is perpendicular to *π*(C_6_H_4_Y-*p*). Characteristic points of *δ*(Se: **2**)_SCS_ are as follows.

1. Large upfield shifts (−21 to −6 ppm) are observed for Y = NMe_2_, OMe, Me, F, Cl, and Br, relative to Y = H.
2. Downfield shifts (3 ppm to 9 ppm) are brought by Y = CN and NO_2_, where the magnitude by Y = CN is larger than that by NO_2_.
3. *δ*(Se: **2**)_SCS_ brought by Y = COOEt is negligible.

While *δ*(Se: **1**)_SCS_ is in a range of −23 < *δ*(Se)_SCS_ < 33 ppm, *δ*(Se: **2**)_SCS_ is −21 < *δ*(Se)_SCS_ < 9 ppm. Y of both donors and acceptors operate well on *δ*(Se: **1**)_SCS_ , whereas only Y of donors do well on *δ*(Se: **2**)_SCS_.

**Figure 1:**
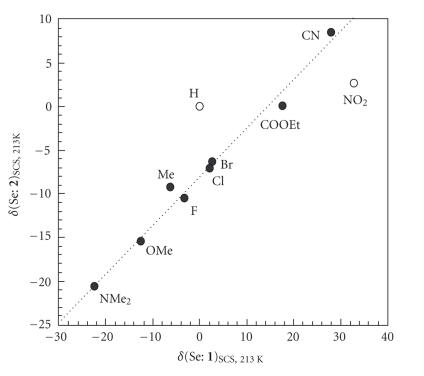
Plot of *δ*(Se: **2**)_SCS, 213 k_ versus *δ*(Se: **1**)_SCS, 213 k_.

*δ*(Se: **2**)_SCS_ are plotted versus those of *δ*(Se: **1**)_SCS_. Figure 1 shows the results. Indeed, it emphasizes the difference in the characters between *δ*(Se: **1**)_SCS_ and *δ*(Se: **2**)_SCS_, but most of *δ*(Se: **2**)_SCS_ seem to correlate well with *δ*(Se: **1**)_SCS_, as shown by a dotted line (*a* = 0.58). Two points corresponding to Y = H and NO_2_ deviate upside and downside from the line, respectively. Namely, points for **2** with Y of non-H are more downside (upfield) than expected from *δ*(Se: **1a**)_SCS_ and *δ*(Se: **2a**)_SCS_, especially for *δ*(Se: **2j**)_SCS_.

Why are such peculiar behaviors observed in **1** and **2**, caused by the orientational effect of the aryl group? The mechanism is elucidated based on the QC calculations performed on **4**–**6**, assuming **pl** and **pd** for each.

### Observed *δ*(Se) versus calculated *σ^t^*(Se)

**Figure 2:**
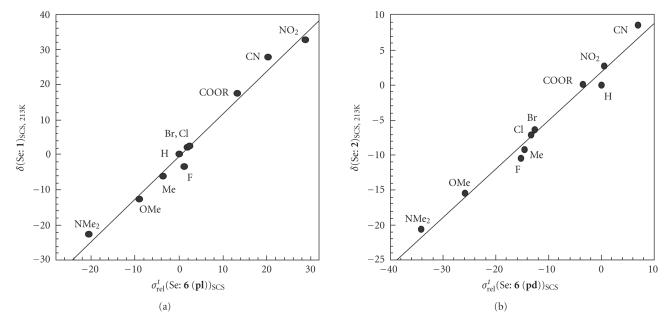
Plots of (a) *δ*(Se: **1**)_SCS, 213 K_ versus *σ^t^*_rel_(Se: **6** (**pl**))_SCS_ and (b) *δ*(Se: **2**)_SCS, 213 K_ versus *σ^t^*_rel_(Se: **6** (**pd**))_SCS_.

The *δ*(Se)_SCS_ values of **1** and **2** are plotted versus *σ^t^*_rel_(Se)_SCS_ of **4** (**pl**)–**6** (**pl**) and **4** (**pd**)–**6** (**pd**), respectively, (Table 1). Good correlations are obtained as shown in Table 2 (entries 5–10). The *r* values become larger in an order of **4**(**pl**) < **5**(**pl**) ≦ **6**(**pl**) for **1** and in an order of **5**(**pd**) < **4**(**pd**) ≈ **6**(**pd**) for **2**. Namely, observed *δ*(Se: **1**)_SCS_ and *δ*(Se: **2**)_SCS_ are reproduced by *σ^t^*_rel_(Se: **6** (**pl**))_SCS_ and *σ^t^*_rel_(Se: **6** (**pd**))_SCS_, respectively, in most successfully. Figure 2 exhibits the plots for (a) **1** versus **6** (**pl**) and (b) **2** versus **6** (**pd**). The correlations are given in Table 2 (entries 7 and 10). The results demonstrate that the characters of *δ*(Se)_SCS_ observed in **1** originate from the planar structure and those in **2** from the characteristic structure, where Se−C_Atq_ in *p*-YC_6_H_4_SeAtq is perpendicular to the *p*-YC_6_H_4_ plane.

How does such orientational effect arise from the structures? How does the electronic property of Y affect on *δ*(Se) of **1** and **2**? *σ^p^*(Se) of **4**–**6** are analyzed next.

### Orientational effect in **4a**–**6a**

*σ*(Se) of **4**–**6** shown in Tables 3–5 are examined. *σ^p^*(Se) and *σ^t^*(Se) of **4a** (**pd**) are evaluated to be larger (more upfield) than those of **4a** (**pl**) by 43 ppm and 46 ppm, respectively, which correspond to the orientational effect caused by Ph in **4a**.^7^ The inverse orientational effect is predicted for **5a**. *σ^p^*(Se) and *σ^t^*(Se) of **5a** (**pd**) are smaller than those of **5a** (**pl**) by 41 ppm and 49 ppm, respectively. While *σ^p^*(Se) and *σ^t^*(Se) of **5a** (**pl**) are smaller than those of **4a** (**pl**) by 90 ppm and 83 ppm, respectively, the values of **5a** (**pd**) are smaller than those of **4a** (**pd**) by 174 ppm and 178 ppm, respectively. The differences are −84 ppm and −95 ppm, respectively, which also correspond to the differences in the orientational effect of the Ph group between **5a** and **4a**, respectively. The more effective contribution to downfield shifts by the Se−C_Me_ bond in **5a** (**pd**), relative to **5a** (**pl**), must be responsible for the results. The orientational effect cannot be discussed for **6a** of the *Cs* symmetry with Y = H.

What mechanism is operating in the Y dependence? *σ^p^*(Se) of **4**–**6** in **pl** and **pd** are analyzed next.

### Y dependence in **4**–**6**

**Figure 3:**
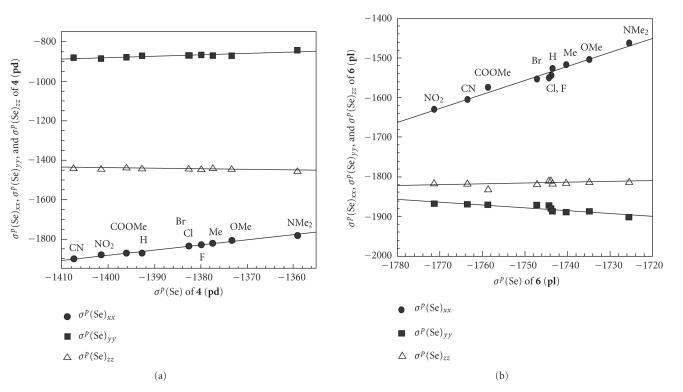
Plots of *σ^p^*(Se)_*xx*_ (●), *σ^p^*(Se)_*yy*_ (■), and *σ^p^*(Se)_*zz*_ (Δ) versus *σ^p^*(Se): (a) for **4** (**pd**) and (b) for **6** (**pl**).

To get an image in the behavior of *σ^p^*(Se)_*xx*_, *σ^p^*(Se)_*yy*_, and *σ^p^*(Se)_*zz*_ of **4**–**6**, the values are plotted versus *σ^p^*(Se). Figure 3 shows the plots for **4** (**pd**) and **6** (**pl**). The correlations in **4** (**pd**) are linear and both *σ^p^*(Se)_*xx*_ and *σ^p^*(Se)_*yy*_ increase along with *σ^p^*(Se). The plot for **5** (**pd**) is similar to that for **4** (**pd**), although not shown. In the case of **6** (**pl**), the correlations are linear but the slope for *σ^p^*(Se)_*yy*_ is inverse to that for *σ^p^*(Se)_*xx*_. The plots of *σ^p^*(Se)_*xx*_ and *σ^p^*(Se)_*yy*_ do not give smooth lines for **4** (**pl**), **5** (**pl**), and **6** (**pd**). However, the slopes for *σ^p^*(Se)_*zz*_ are very smooth and the magnitudes are very close to 1.0 for all cases in **4**–**6**.

**Figure 4:**
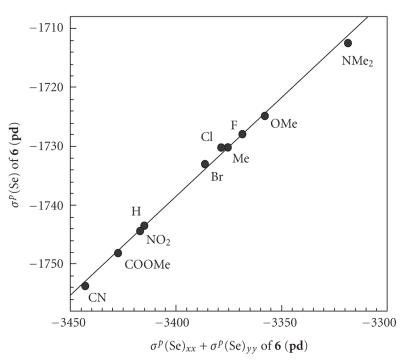
Plot of *σ^p^*(Se) versus *σ^p^*(Se)_*xx*_ + *σ^p^*(Se)_*yy*_ for **6** (**pd**).

To clarify the behavior of *σ^p^*(Se) in **4**–**6**, *σ^p^*(Se) are plotted versus (*σ^p^*(Se)_*xx*_ + *σ^p^*(Se)_*yy*_).^8^ Excellent to good correlations are obtained in all cases as collected in Table 2 (entries 11–16). Figure 4 exhibits the plot of *σ^p^*(Se) for **6** (**pd**), for example. The correlation constants (*a*) are 0.31–0.37, which are very close to one third. The results exhibit that (*σ^p^*(Se)_*xx*_+*σ^p^*(Se)_*yy*_) determines *σ^p^*(Se) of **4**–**6** effectively (cf: (2)). The observations led us to establish the mechanism of Y dependence in **4**–**6**.

### Mechanism of Y dependence

**Scheme 3:**
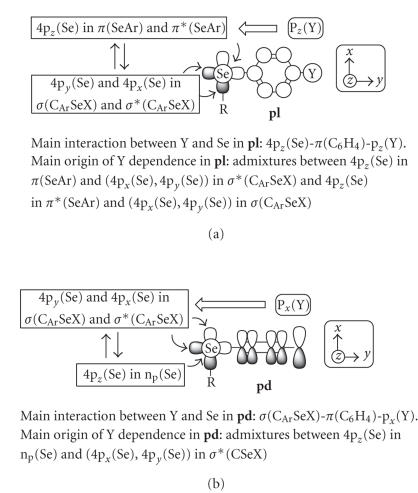
Mechanisms of Y dependence. Outline allows exhibit the effect of p(Y) on 4p(Se) and double allows show the main admixtures to originate *δ*(Se): (a) in **pl** and (b) in **pd**.

The mechanism of Y dependence in **4**–**6** is elucidated by exemplifying **4**. As shown in Scheme 2, the main interaction between Se and Y in **4** (**pl**) is the 4p_*z*_(Se)-*π*(C_6_H_4_)-p_*z*_(Y) type, which modifies the contributions of 4p_*z*_(Se) in *π*(SeC_6_H_4_Y) and π*(SeC_6_H_4_Y). Since (*σ^p^*(Se)_*xx*_ + *σ^p^*(Se)_*yy*_) controls *σ^p^*(Se) of **4** (**pl**) effectively, admixtures between 4p_*z*_(Se) in modified *π*(SeC_6_H_4_Y) and π*(SeC_6_H_4_Y) with 4p_*y*_(Se) and 4p_x_(Se) in *σ*(C_Ar_SeH) and σ*(C_Ar_SeH) must originate the Y dependence mainly when a magnetic field is applied.^9^ Since *σ^p^*_*zz,N*_ contains the ^*L_z,N_* operator, *σ^p^*_*zz,N*_ arises from admixtures between atomic p_*x*_ and p_*y*_ orbitals of *N* in various molecular orbitals. When a magnetic field is applied on a selenium compound, mixings of unoccupied molecular orbitals (MO's; *ψ*_*i*_) into occupied orbital MO's (*ψ*_*i*_) will occur. Such admixtures generate *σ^p^*_*zz,N*_ if *ψ*_*i*_ and *ψ*_*j*_ contain p_*x*_ and p_*y*_ of *N*, for example. *σ^p^*_*xx,N*_ and *σ^p^*_*yy,N*_ are also understood similarly. Consequently, Y of both donors and acceptors are effective for the Y dependence in **4** (**pl**). Scheme 3(a) shows the mechanism for **pl**.

In the case of **4** (**pd**), 4p_*z*_(Se) remains in n_*p*_(Se) in the almost pure form.^10^ The *σ*(C_Ar_SeH)-*π*(C_6_H_4_)-p_*x*_(Y) interaction occurs instead, which modifies the contributions of 4p_x_(Se) and 4p*_y_*(Se) in *σ*(C_Ar_SeH) and σ*(C_Ar_SeH) (see Scheme 2). (*σ^p^*(Se)_*xx*_ + *σ^p^*(Se)_*yy*_) determines effectively *σ^p^*(Se) of **4** (**pd**). Therefore, Y dependence of **4** (**pd**) originates mainly from admixtures between 4p_*z*_(Se) in n_*p*_(Se) and 4p_*x*_(Se) and 4p_*y*_(Se) in modified σ*(C_Ar_SeH) since n_*p*_(Se) of 4p_*z*_(Se) is filled with electrons. Consequently, Y dependence in **4** (**pd**) must be more sensitive to Y of donors, which is a striking contrast to the case of **4** (**pl**). Scheme 3(b) summarizes the mechanism for **pd**.

The mechanisms proposed for **4** (**pl**) and **4** (**pd**) must be applicable to **5** and **6**. The expectations are just observed in *δ*(Se: **1**)_SCS_ and *δ*(Se: **2**)_SCS_.

### Applications of *δ*(Se: **1**) and *δ*(Se: **2**) as the standards

Odom made a lot of effort to explain *δ*(Se) of **7** based on the electronic effect of Y [13]. However, the attempt was not successful: *δ*(Se: **7**) were not correlated well with *δ*(Se: **5**). How are *δ*(Se) of *p*-YC_6_H_4_SeR interpreted based on the orientational effect? Our explanation for the relationship between *δ*(Se) of *p*-YC_6_H_4_SeR and the structures is as follows.

**Figure 5:**
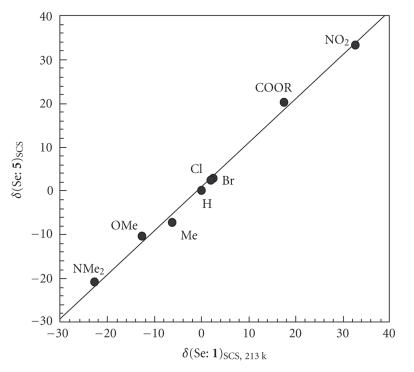
Plot of *δ*(Se: **5**)_SCS_ versus *δ*(Se: **1**)_SCS, 213 k_.

**Figure 6:**
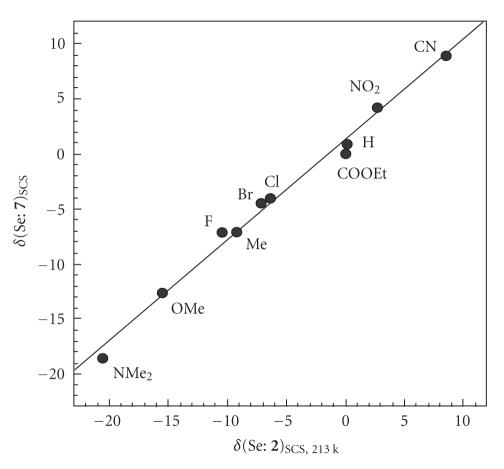
Plot of *δ*(Se: **7**)_SCS_ versus *δ*(Se: **2**)_SCS, 213 k_.

Figure 5 shows the plot of *δ*(Se: **5**)_SCS_ measured in CDCl_3_ [19] versus *δ*(Se: **1**)_SCS, 213 K_ and the correlation is given in Table 2 (entry 17: *r* = 0.997). The correlation coefficient is excellent when *δ*(Se: **5**)_SCS_ measured in neat is plotted versus *δ*(Se: **1**)_SCS, 213 K_ (entry 18 in Table 2: *r* = 0.999). These observations must be the results of the Se−C_Me_ bond in **5** being on the *p*-YC_6_H_4_ plane in solutions for all Y examined, under the conditions. On the other hand, *δ*(Se: **7**)_SCS_ do not correlate with *δ*(Se: **1**)_SCS, 213 K_. Instead, they correlate well with *δ*(Se: **2**)_SCS, 213 K_ (entry 19 in Table 2: *r* = 0.995). Figure 6 shows the plot. The results are rationally explained by assuming that the Se−C_O_ bond in **7** is perpendicular to the *p*-YC_6_H_4_ plane in solutions for all Y examined, under the conditions.

*δ*(Se)_SCS_ of **6** [19] and **8** [8] are similarly plotted versus *δ*(Se: **1**)_SCS, 213 K_. They give good correlations, although the *r* values become poorer relative to that for **5** (entries 20 and 21 in Table 2). The reason would be the equilibrium of **pl** with **pd** for some Y in **6** and **8**, may be Y of donors.

*δ*(Se)_SCS_ of **9** are also plotted versus *δ*(Se: **1**)_SCS, 213 K_. The correlations are excellent (entry 22 in Table 2: *r* = 0.999). It is worthwhile to comment that the energy lowering effect by Se_4_ 4c–6e in **9** fixes the conformation **9** (**pl**, **pl**) for both *p*-YC_6_H_4_Se groups in solutions for all Y examined, under the conditions [50].

It is demonstrated that sets of *δ*(Se: **1**) and *δ*(Se: **2**) proposed in this work can be the standards for **pl** and **pd**, respectively, when *δ*(Se) of aryl selenides are analyzed based on the orientational effect.

## CONCLUSION

The orientational effect is empirically established by the Y dependence on *δ*(Se: **1**) and *δ*(Se: **2**). The Y dependence observed in **1** and **2** is demonstrated by *σ^t^*(Se) calculated for **4**–**6** with the DFT-GIAO method. While *σ^t^*(Se) of **4a** (**pl**) is predicted to be more negative than that of **4a** (**pd**) by 46 ppm, *σ^t^*(Se) of **5a** (**pl**) is evaluated to be larger than that of **5a** (**pd**) by 49 ppm, which corresponds to the orientational effect by the Ph group in **4a** and **5a**, respectively. Excellent to good correlations are obtained in the plots of *σ^p^*(Se) versus (*σ^p^*(Se)_*xx*_+*σ^p^*(Se)_*yy*_) for **4**–**6** in **pl** and **pd**. It is demonstrated that (*σ^p^*(Se)_*xx*_ + *σ^p^*(Se)_*yy*_) effectively controls *σ^p^*(Se) of **4**–**6** in **pl** and **pd**.

The mechanisms of the Y dependence are proposed based on the magnetic perturbation theory. The main interaction in **pl** is the n_*p*_(Se)-*π*(C_6_H_4_)-p_*z*_(Y) conjugation. Y dependence in **pl** occurs through admixtures of 4p_*z*_(Se) in modified *π*(SeC_6_H_4_Y) and π*(SeC_6_H_4_Y) with 4p_*x*_(Se) and 4p_*y*_(Se) in *σ*(CSeX) and σ*(CSeX) (X = H or C). The main interaction in **pd** is the *σ*(CSeX)-*π*(C_6_H_4_)-p_*x*_(Y) type, which modifies both *σ*(C_Ar_SeH) and σ*(C_Ar_SeH). The Y dependence in **pd** mainly originates from admixtures of 4p_*z*_(Se) in n_*p*_(Se) with 4p_*x*_(Se) and 4p_*y*_(Se) in modified σ*(CSeX) since n_*p*_(Se) of 4p_*z*_(Se) is filled with electrons. Therefore, Y of both donors and acceptors are effective in **pl**, whereas Y of donors are more effective in **pd**. The expectations are just observed in **1** and **2**. Sets of *δ*(Se: **1**) and *δ*(Se: **2**) can be used as the standards for **pl** and **pd**, respectively, when *δ*(Se) of aryl selenides are analyzed.

The effect of R in ArSeR is also important, which is in progress. The results will be discussed elsewhere, together with the applications of the method.

## EXPERIMENTAL

NMR spectra were recorded at 25°C on a JEOL JNM-AL 300 spectrometer (^1^ H, 300 MHz; ^13^ C, 75.45 MHz; ^77^ Se, 57.25 MHz). The ^1^ H, ^13^ C, and ^77^ Se chemical shifts are given in parts per million relative to those of Me_4_Si, internal CDCl_3_ in the solvent, and external MeSeMe, respectively.

### Preparation of compounds

**1a**–**1j** were prepared by the reactions of anthracenylgrignard reagents with arylselanylbromides and/or aromatic diazonium salts with anthracenylselenolates as the case of **3** [14]. **2a**–**2j** were prepared by the reactions of 8-chloroanthraquione and arylselenolates with CuI as described earlier [51]. Elementary analyses for the compounds were satisfactory to those calculated within ±0.3% accuracy. ^1^ H, ^13^ C, and ^77^ Se NMR chemical shifts of the compounds rationalize the structures.

### MO calculations

Quantum chemical (QC) calculations were performed using a Silent-SCC T2 (Itanium2) computer with the 6-311+G(3df) basis sets for Se and 6-311+G(3d,2p) for other nuclei of the Gaussian 03 program [47]. Calculations are performed on **4**–**6** in **pl** and **pd** at the density functional theory (DFT) level of the Becke three parameter hybrid functionals combined with the Lee-Yang-Parr correlation functional (B3LYP). Absolute magnetic shielding tensors of Se nuclei (*σ*(Se)) are calculated based on the gauge-independent atomic orbital (GIAO) method, applying on the optimized structures with the same method.

Structures of **1a**–**3a** in various conformers are also optimized, containing frequency analysis, with the B3LYP/6-311+G(d,p) method.

## Figures and Tables

**Table 1:** Observed *δ*(Se)_SCS_ of **1** and **2** and calculated *σ^t^*_rel_(Se)_SCS_ for **4**–**6** in **pl** and **pd** ^(a,b)^.

Compd	*T*	NMe_2_	OMe	Me	H	F	Cl	Br	CO_2_R^(c)^	CN	NO_2_
	[K]	(**b**)	(**c**)	(**d**)	(**a**)	(**e**)	(**f**)	(**g**)	(**h**)	(**i**)	(**j**)

**1**	213	−22.7	−12.7	−6.3	0.0 (245.3)	−3.3	1.9	2.4	17.4	27.7	32.7
**1**	297	−21.0	−12.2	−6.6	0.0 (249.0)	−3.6	1.5	1.6	16.2	26.2	30.4
**1**	333	−21.3	−12.7	−6.8	0.0 (250.6)	−3.9	1.0	1.2	15.2	24.8	29.0
**2**	213	−20.6	−15.5	−9.2	0.0 (511.4)	−10.5	−7.1	−6.4	0.1	8.5	2.7
**2**	297	−19.6	−15.0	−9.0	0.0 (512.3)	−10.2	−7.1	−6.4	0.0	8.2	2.5
**2**	333	−19.5	−15.0	−9.1	0.0 (512.5)	−10.3	−7.2	−6.7	−0.3	7.9	2.2
**4** (**pl**)	—	−36.4	−18.0	−8.2	0.0 (87.0)	−1.6	1.7	−1.8	14.3	29.8	33.7
**4** (**pd**)	—	−35.9	−23.0	−15.6	0.0 (41.3)	−11.8	−9.1	−8.7	1.0	16.8	10.0
**5** (**pl**)	—	−23.9	−8.2	−8.0	0.0 (169.7)	2.1	4.7	7.2	24.6	29.7	43.8
**5** (**pd**)	—	−34.9	−21.2	−16.7	0.0 (219.1)	−14.1	−11.8	−12.6	3.0	13.4	6.6
**6** (**pl**)	—	−20.5	−9.0	−3.7	0.0 (398.8)	1.1	1.9	2.3	13.1	20.2	28.6
**6** (**pd**)	—	−34.2	−25.8	−14.6	0.0 (398.8)	−15.2	−13.3	−12.6	−3.4	7.0	0.5

^(a)^ *δ*(Se)_SCS_ are given for **1** and **2**, together with *δ*(Se) for **1** a and **2** a in parenthesis, measured in chloroform-*d*.

^(b)^ *σ^t^*_rel_(Se)_SCS_ are given for **4**−**6**, together with *σ^t^*_rel_(Se) for **4a**−**6a** in parenthesis, calculated according to (3), where *σ^t^*(Se) of **4**−**6** in **pl** and **pd** are given in Tables 3–5, respectively, and *σ^t^*(Se: MeSeMe) = 1650.4 ppm.

^(c)^ R = Et for **1** and **2** and R = Me for **4**−**6**.

**Table 2:** Correlations of *δ*(Se)_SCS_ for **1** and **2** and *σ*(Se) for **4**−**6**, together with *δ*(Se)_SCS_ for **5**−**9** ^(a)^.

Entry	Correlation	*a*	*b*	*r*	*n* ^(b)^

1	*δ*(Se: **1**)_SCS, 297 K_ vs *δ*(Se: **1**)_SCS, 213 K_	0.940	−0.3	1.000	10
2	*δ*(Se: **1**)_SCS, 333 K_ vs *δ*(Se: **1**)_SCS, 213 K_	0.916	−0.8	1.000	10
3	*δ*(Se: **2**)_SCS, 297 K_ vs *δ*(Se: **2**)_SCS, 213 K_	0.957	−0.1	1.000	10
4	*δ*(Se: **2**)_SCS, 333 K_ vs *δ*(Se: **2**)_SCS, 213 K_	0.946	−0.3	1.000	10
5	*δ*(Se: **1**)_SCS, 213 K_ vs *σ* ^rel^(Se: **4** (**pl**))_SCS_	0.823	2.6	0.986	10
6	*δ*(Se: **1**)_SCS, 213 K_ vs *σ* ^rel^(Se: **5** (**pl**))_SCS_	0.845	−2.1	0.990	10
7	*δ*(Se: **1**)_SCS, 213 K_ vs *σ* ^rel^(Se: **6** (**pl**))_SCS_	1.218	−0.4	0.991	10
8	*δ*(Se: **2**)_SCS, 213 K_ vs *σ* ^rel^(Se: **4** (**pd**))_SCS_	0.562	−1.5	0.990	10
9	*δ*(Se: **2**)_SCS, 213 K_ vs *σ* ^rel^(Se: **5** (**pd**))_SCS_	0.599	−0.5	0.988	10
10	*δ*(Se: **2**)_SCS, 213 K_ vs *σ* ^rel^(Se: **6** (**pd**))_SCS_	0.691	1.9	0.990	10
11	*σ^p^*(Se) vs (*σ^p^*(Se)_*xx*_ + *σ^p^*(Se)_*yy*_) in **4** (**pl**)	0.339	−547.8	0.982	10
12	*σ^p^*(Se) vs (*σ^p^*(Se)_*xx*_ + *σ^p^*(Se)_*yy*_) in **5** (**pl**)	0.367	−461.8	0.999	10
13	*σ^p^*(Se) vs (*σ^p^*(Se)_*xx*_ + *σ^p^*(Se)_*yy*_) in **6** (**pl**)	0.350	−546.7	0.990	10
14	*σ^p^*(Se) vs (*σ^p^*(Se)_*xx*_ + *σ^p^*(Se)_*yy*_) in **4** (**pd**)	0.309	−547.0	0.998	10
15	*σ^p^*(Se) vs (*σ^p^*(Se)_*xx*_ + *σ^p^*(Se)_*yy*_) in **5** (**pd**)	0.345	−517.4	0.994	10
16	*σ^p^*(Se) vs (*σ^p^*(Se)_*xx*_ + *σ^p^*(Se)_*yy*_) in **6** (**pd**)	0.335	−598.5	0.998	10
17	*δ*(Se: **5**)_SCS_ ^(c)^ vs *δ*(Se: **1**)_SCS, 213 K_	0.997	1.0	0.997	8
18	*δ*(Se: **5**)_SCS_ ^(d)^ vs *δ*(Se: **1**)_SCS, 213 K_	0.952	0.1	0.999	7
19	*δ*(Se: **7**)_SCS_ vs *δ*(Se: **2**)_SCS, 213 K_	0.909	1.3	0.995	10
20	*δ*(Se: **6**)_SCS_ vs *δ*(Se: **1**)_SCS, 213 K_	0.804	−3.3	0.991	7
21	*δ*(Se: **8**)_SCS_ vs *δ*(Se: **1**)_SCS, 213 K_	0.691	−1.7	0.981	9
22	*δ*(Se: **9**)^*c*^_SCS_ vs *δ*(Se: **1**)_SCS, 213 K_	0.870	−1.3	0.999	7

^(a)^The constants (*a, b, r*) are defined by *y* = *ax* + *b* (*r*: correlation coefficient).

^(b)^The number of data used in the correlation. ^(c)^Reference [19] at neat. ^(d)^Reference [11] in CDCl_3_.

**Table 3:** Calculated absolute shielding tensors (*σ*(Se)) of **4**, containing various Y^(a)^.

Y	*σ^d^*(Se)	*σ^p^*(Se)_*xx*_	*σ^p^*(Se)_*yy*_	*σ^p^*(Se)_*zz*_	*σ^p^*(Se)	*σ^t^*(Se)

**4** (**pl**)						

H	2999.5	−1571.7	−1042.3	−1694.2	−1436.1	1563.4
NMe_2_	3006.4	−1676.1	−862.4	−1681.4	−1406.7	1599.8
OMe	3004.7	−1823.5	−757.0	−1689.5	−1423.3	1581.4
Me	3002.4	−1760.2	−848.1	−1684.2	−1430.8	1571.6
F	3001.4	−1800.4	−833.2	−1675.7	−1436.4	1565.0
Cl	3003.8	−1777.8	−868.7	−1680.0	−1442.2	1561.7
Br	3008.7	−1883.4	−745.3	−1701.6	−1443.4	1565.2
COOMe	3010.0	−1469.6	−1197.4	−1715.7	−1460.9	1549.1
CN	3002.1	−1829.1	−889.8	−1686.5	−1468.5	1533.6
NO_2_	3004.9	−1836.6	−905.8	−1683.2	−1475.2	1529.7

**4** (**pd**)						

H	3001.9	−1870.9	−869.9	−1437.6	−1392.8	1609.1
NMe_2_	3004.1	−1782.2	−842.4	−1452.8	−1359.1	1645.0
OMe	3005.4	−1805.2	−871.3	−1443.6	−1373.4	1632.1
Me	3002.2	−1821.7	−871.0	−1439.8	−1377.5	1624.7
F	3000.8	−1829.8	−866.2	−1443.7	−1379.9	1620.9
Cl	3000.8	−1834.5	−870.2	−1442.8	−1382.5	1618.2
Br	3000.5	−1835.5	−870.5	−1442.1	−1382.7	1617.8
COOMe	3004.2	−1872.6	−879.2	−1436.5	−1396.1	1608.1
CN	2999.9	−1901.0	−881.6	−1440.1	−1407.6	1592.3
NO_2_	3000.7	−1877.7	−884.4	−1442.8	−1401.6	1599.1

^(a)^ Structures are optimized with the 6-311+G(3df) basis sets for Se and 6-311+G(3d,2p) basis sets for other nuclei at the DFT (B3LYP) level, assuming **pl** and **pd** for each of Y [47]. *σ*(Se) are calculated based on the DFT-GIAO method with the same methods.

**Table 4:** Calculated absolute shielding tensors (*σ*(Se)) of **5**, containing various Y^(a)^.

Y	*σ^d^*(Se)	*σ^p^*(Se)_*xx*_	*σ^p^*(Se)_*yy*_	*σ^p^*(Se)_*zz*_	*σ^p^*(Se)	*σ^t^*(Se)

**5** (**pl**)						

H	3006.5	−1893.4	−999.0	−1684.9	−1525.8	1480.7
NMe_2_	3007.7	−1645.4	−1194.5	−1669.5	−1503.1	1504.6
OMe	3007.4	−1741.5	−1136.8	−1677.1	−1518.4	1488.9
Me	3008.0	−1815.2	−1064.7	−1678.0	−1519.3	1488.7
F	3006.2	−1911.7	−990.8	−1680.6	−1527.7	1478.6
Cl	3006.7	−1639.8	−1269.8	−1682.4	−1530.7	1476.0
Br	3008.1	−1768.8	−1156.2	−1679.0	−1534.7	1473.5
COOMe	3009.6	−1840.5	−1132.8	−1687.1	−1553.5	1456.1
CN	3006.6	−1601.6	−1377.0	−1688.1	−1555.6	1451.0
NO_2_	3007.0	−1800.0	−1220.1	−1690.0	−1570.1	1436.9

**5** (**pd**)						

H	2998.0	−1956.8	−1086.4	−1656.9	−1566.7	1431.3
NMe_2_	3003.5	−1889.2	−1062.0	−1660.9	−1537.3	1466.2
OMe	3004.1	−1938.6	−1059.6	−1656.6	−1551.6	1452.5
Me	2999.8	−1908.0	−1090.1	−1657.2	−1551.8	1448.0
F	2998.1	−1916.6	−1077.7	−1663.9	−1552.8	1445.4
Cl	2999.3	−1925.8	−1078.6	−1664.3	−1556.2	1443.1
Br	3001.0	−1930.0	−1077.7	−1663.5	−1557.1	1443.9
COOMe	3006.4	−2017.8	−1057.8	−1658.7	−1578.1	1428.3
CN	2998.0	−1995.5	−1076.6	−1668.2	−1580.1	1417.9
NO_2_	2999.5	−1977.4	−1075.9	−1671.0	−1574.7	1424.7

^(a)^Structures are optimized with the 6-311+G(3df) basis sets for Se and 6-311+G(3d,2p) basis sets for other nuclei at the DFT (B3LYP) level, assuming **pl** and **pd** for each of Y [47]. *σ*(Se) are calculated based on the DFT-GIAO method with the same methods.

**Table 5:** Calculated absolute shielding tensors (*σ*(Se)) of **6**, containing various Y^(a)^.

Y	*σ^d^*(Se)	*σ^p^*(Se)_*xx*_	*σ^p^*(Se)_*yy*_	*σ^p^*(Se)_*zz*_	*σ^p^*(Se)	*σ^t^*(Se)

**6** (**pl**)						

H	2995.1	−1527.4	−1887.5	−1815.6	−1743.5	1251.6
NMe_2_	2997.7	−1462.8	−1902.3	−1811.6	−1725.5	1272.1
OMe	2995.5	−1504.1	−1887.4	−1813.2	−1734.9	1260.6
Me	2995.6	−1517.7	−1888.8	−1814.2	−1740.3	1255.3
F	2994.5	−1544.4	−1879.4	−1808.1	−1743.9	1250.5
Cl	2994.1	−1550.2	−1873.8	−1809.2	−1744.4	1249.7
Br	2996.5	−1553.1	−1871.1	−1817.4	−1747.2	1249.3
COOMe	2997.2	−1574.5	−1871.7	−1830.0	−1758.7	1238.5
CN	2994.8	−1605.6	−1869.2	−1815.6	−1763.5	1231.4
NO_2_	2994.4	−1630.8	−1867.8	−1815.7	−1771.4	1223.0

**6** (**pd**)						

H	2995.1	−1887.5	−1527.4	−1815.6	−1743.5	1251.6
NMe_2_	2998.3	−1787.3	−1531.6	−1818.6	−1712.5	1285.8
OMe	3002.2	−2044.1	−1313.9	−1816.4	−1724.8	1277.4
Me	2996.4	−1843.1	−1532.7	−1814.8	−1730.2	1266.2
F	2994.8	−1851.2	−1517.7	−1815.0	−1728.0	1266.8
Cl	2995.1	−1859.5	−1519.1	−1812.1	−1730.2	1264.9
Br	2997.2	−1871.4	−1514.8	−1812.8	−1733.0	1264.2
COOMe	3003.2	−2085.4	−1341.9	−1817.2	−1748.2	1255.1
CN	2998.5	−2132.1	−1310.6	−1818.8	−1753.8	1244.6
NO_2_	2995.5	−1914.6	−1502.6	−1816.1	−1744.4	1251.1

^(a)^Structures are optimized with the 6-311+G(3df) basis sets for Se and 6-311+G(3d,2p) basis sets for other nuclei at the DFT (B3LYP) level, assuming **pl** and **pd** for each of Y [47]. *σ*(Se) are calculated based on the DFT-GIAO method with the same methods.

**Table 6:** Observed *δ*(Se)_SCS_ reported for **5**−**9**.

Compd	NMe_2_	OMe	Me	H	F	Cl	Br	CO_2_R^(a)^	CN	NO_2_
	(**b**)	(**c**)	(**d**)	(**a**)	(**e**)	(**f**)	(**g**)	(**h**)	(**i**)	(**j**)

**5** ^(b)^	−20.8	−10.4	−7.2	0.0 (207.8)	—	2.5	2.8	20.1	—	33.4
**5** ^(c)^	—	−12.5	−5.9	0.0 (202.0)	−2.0	1.6	—	16.1	—	31.4
**6** ^(b)^	—	−15.5	−8.6	0.0 (423.6)	—	−1.7	−1.3	9.7	—	22.7
**7** ^(d)^	−18.6	−12.6	−7.1	0.0 (641.5)	−7.1	−4.5	−4.1	0.8	8.9	4.2
**8** ^(e)^	—	−12.0	−7.8	0.0 (320.8)	−2.5	0.2	0.9	8.6	21.0	18.0
**9** ^(f)^	—	−9.8	−6.6	0.0 (434.3)	—	−2.7	−1.9	8.1	—	19.6

^(a)^ R = Me for **5** and R = Et for **6**−**9**. ^(b)^Reference [19]. ^(c)^Reference [11] at neat. ^(d)^Reference [13]. ^(e)^Reference [8]. ^(f)^Reference [15, 16].

## Footnotes

^1^ The contribution of relativistic terms has been pointed out for heavier atoms, but the perturbation would be small for the selenium nucleus.

^2^ This decomposition includes small arbitrariness due to the coordinate origin dependence, though it does not damage our chemical analyses and insights into the ^77^ Se NMR spectroscopy.

^3^ The 0.050 M CDCl_3_ solutions were used for NMR measurements. However, the concentrations would be lower for the compounds of low solubility, such as **1j** and **2j**, especially at 213 K.

^4^ When the axes from the Gaussian 03 program [47] are not the same as those shown in Scheme 3, axes are interchanged so as to be those in Scheme 3 for convenience of discussion, if possible.

^5^ Gaussian 03 (Revision B.05).

^6^ The torsional angle of *φ* = C_*o*_C_*i*_SeH in **4** (**pd**) is fixed at 90.0° if **4** (**pd**) is not the *Cs* symmetry (eg, Y = COOMe and OMe). Similarly, those of *φ* = C_*o*_C_*i*_SeC_Me_ and *φ* = C_*i*_SeC_Me_H in **5** (**pd**) are fixed at 90.0° and 180°, respectively, and those of *φ* = C_*o*_C_*i*_SeC_*i'*_ and *φ* = C_*i*_SeC_*i'*_C_*o'*_ in **6** (**pd**) are fixed at 90.0° and 0°, respectively, when the conformers are not the *Cs* symmetry.

^7^ The DFT shieldings are deshielded in general, due to the underestimation of the orbital energy differences, which lead to the overestimation of the *σ^p^*(Se) [48]. MP2 calculations are also performed on **4a** (**pl**), **4a** (**pd**), **5a** (**pl**), and **5a** (**pd**). The geometries are optimized with the MP2/6-311+G(3d,2p) method. *σ^t^*(Se) are calculated with the MP2-GIAO method, employing the 6-311+G(2d,p) basis sets. The results are as follows (in ppm): (*σ^t^*(Se: **4a** (**pl**)), *σ^t^*(Se: **4a** (**pd**))) = (1827.3, 1865.5) and (*σ^t^*(Se: **5a** (**pl**)), *σ^t^*(Se: **5a** (**pd**))) = (1761.0, 1708.7). *σ^t^*(Se: **4a** (**pl**)) is evaluated to be more downfield than *σ^t^*(Se: **4a** (**pd**)) by 38 ppm, whereas *σ^t^*(Se: **5a** (**pl**)) is evaluated to be more upfield than *σ^t^*(Se: **5a** (**pd**)) by 52 ppm. The results support the orientational effects evaluated at the DFT level for **4a** and **5a**, although the basis sets are not the same.

^8^ *σ^p^*(Se)_*zz*_ is almost constant in the change of Y for both **pl** and **pd** in **4**–**6**. The small Y-dependence of *σ^p^*(Se)_*zz*_ is reasonably explained through the main interaction of the 4p_*z*_(Se)-*π*(C_6_H_4_)-p_*z*_(Y) type in **pl**, where 4p_*x*_(Se) and 4p_*y*_(Se) do not take part in the interaction directly. The main interaction in **pd** is the *σ*(C_Ar_SeX)-*π*(C_6_H_4_)-p_*x*_(Y) (X = H or C) type, which modifies the contributions of 4p_*x*_(Se) and 4p_*y*_(Se) in the C_Ar_SeX bonds. However, the results show that the interaction in **pd** affects on *σ^p^*(Se)_*xx*_ and *σ^p^*(Se)_*yy*_ but not on *σ^p^*(Se)_*zz*_.

^9^ *σ^p^* is exactly expressed by Ramsey's equation [49]. While *σ^p^* is evaluated accurately by the CPHF method, it is approximated as *σ^p^_zz,N_* = −(*μ_o_e^2^/2m^2^_e_*) *Σ_i_^occ^* *Σ_j_^unocc^* (*ɛ_j_* − *ɛ_i_*)^−1^ × {< *ψ_i_*|^*L_z_*|*ψ_j_* >< *ψ_j_*|^*L_z,N_* r_*N*_^−3^|*ψ*_*i*_ > + < *ψ*_*i*_|^*L_z,N_* r_N_^−3^|*ψ*_*j*_ >< *ψ*_*j*_|^*L_z_*|*ψ*_*i*_ >}.

^10^ The interactions between n_*p*_(Se) of 4p_*z*_(Se) and phenyl *σ* orbitals in **4a** (**pd**) must be weak due to large energy differences between 4p_*z*_(Se) and the *σ* orbitals. Long distances between them are also disadvantageous.
